# Commentary on the article “Translation, cross-cultural adaptation and validation of the Caregiver Indirect and Informal Care Costs Assessment Questionnaire for end-of-life care into Spanish” by Lamfre et al.

**DOI:** 10.1017/S1478951525101557

**Published:** 2026-01-08

**Authors:** Erik Landfeldt

**Affiliations:** IQVIA, Stockholm, Sweden

Caregiver burden remains a central concern in palliative and supportive care, with informal caregivers (e.g., parents, partners, and other family members) providing indispensable support that is frequently neglected in cost research and economic evaluations. The recent article by Lamfre et al. (2025) represents a significant contribution to the field, offering a rigorously translated, cross-culturally adapted, and validated Spanish version of the Caregiver Indirect and Informal Care Costs Assessment Questionnaire (CIIQ) (Landfeldt et al. 2019) for use in Argentina. The authors are to be congratulated for their meticulous approach and for addressing a critical gap in the measurement of caregiver costs in Spanish-speaking populations. As a robust instrument capturing the multifaceted nature of caregiver burden and its economic impact, the adaptation of the CIIQ will undoubtedly facilitate the design and implementation of comprehensive cost-of-illness studies informing health policy.

The importance of accurately measuring caregiver burden cannot be overstated. Informal caregivers shoulder a substantial proportion of the total care of many diseases, often at great personal and financial sacrifice (Hoefman et al. 2013; Adelman et al. 2014). Instruments like the CIIQ are essential for accurately quantifying and making these contributions visible. Moreover, by enabling the estimation of both indirect (productivity) and informal care costs for caregivers with widely different work statuses and caregiving roles – including varied levels of absenteeism from work, lost productivity while working, and unpaid and paid informal care – based on established methodology (i.e., the opportunity cost and proxy good methods), the CIIQ provides a standardized framework that is both rigorous and practical.

While the translation and cultural adaptation of the CIIQ are exemplary, I would like to offer a friendly methodological observation regarding the psychometric analysis presented. The items in the CIIQ instrument constitute the minimum data elements required to estimate caregiver indirect (productivity) and informal care costs as separate, mutually exclusive subsets of total costs. Each item is necessary for the calculation of these costs according to the opportunity cost and proxy good methods. As such, it is not possible to remove a question and still estimate indirect (productivity) and informal care costs. Similarly, it is not clear how adding a question could improve the estimation of these costs, given the instrument’s design and purpose. The conclusion that “the productivity-related items could benefit from further refinement” may therefore be misleading in the context of cost estimation, as the structure of the questionnaire is dictated by the requirements of the underlying economic methodology rather than by psychometric optimization.

In summary, Lamfre et al. have made a significant addition to the field by providing a culturally adapted and validated tool for the assessment of caregiver costs in Spanish-speaking settings. Their work will help ensure that the economic impact of informal caregiving is appropriately recognized in future research and policy. I encourage continued dialogue on the methodological nuances of cost estimation in caregiver research, and I look forward to further studies that build on this important foundation.
